# Genome-Wide SNPs Provide Insights on the Cryptic Genetic Structure and Signatures of Climate Adaption in *Amorphophallus albus* Germplasms

**DOI:** 10.3389/fpls.2021.683422

**Published:** 2021-07-23

**Authors:** Yong Gao, Si Yin, Honglong Chu, Yanan Zhang, Haibo Wang, Huanhuan Chen, Chao Liu, Dongqin Dai, Lizhou Tang

**Affiliations:** College of Biological Resource and Food Engineering, Center for Yunnan Plateau Biological Resources Protection and Utilization, Qujing Normal University, Qujing, China

**Keywords:** *Amorphophallus albus*, restriction site-associated DNA sequencing, genetic structure, isolation by distance, isolation by environment, environment adaption

## Abstract

Domesticated species represent unique systems in which the evolutionary genomic consequences of intensive selective breeding and adaptation can be thoroughly investigated. *Amorphophallus albus* occurs naturally and is in cultivation throughout the downstream region of the Jinshajiang River in Southwest China. This species is characterised by high konjac glucomannan content, and has been cultivated in China for nearly 2,000 years. To study genetic differentiation and local adaption of *A. albus*, we sampled 13 distinct local cultivated populations of this species. Restriction site-associated DNA sequencing was conducted with 87 samples, resulting in 24,225 SNPs. The population structure analyses suggest two main genetic groups: one in the relatively upstream region, and one downstream. We found evidence of additional sub-structure within the upstream group, demonstrating the statistical power of genomic SNPs in discovering subtle genetic structure. The environmental and geographic factors were all identified as significant in shaping the genetic differentiation of this species. Notably, the proportion of environmental factors was larger than geographic factors in influencing the population genetic patterns of *A. albus*. We also discovered loci that were associated with local adaptation. These findings will help us understand the genetic differentiation of this newly domesticated species, thereby informing future breeding programs of *A. albus*.

## Introduction

Understanding the genetic basis of adaptation has been a long standing goal of evolutionary biology (Barton and Keightley, 2002; Grillo et al., 2016). Domesticated species are excellent biological models for studying the genetic basis of rapid evolutionary response to abiotic selective pressure such as climate (Flori et al., 2019). For example, cultivated crops may face strong selective pressure during the domestication process, resulting in population genomics changes. Therefore, the population genomic resources from crops are extremely valuable in understanding the genomic patterns of microevolution under natural and artificial selection.

**FIGURE 1:**
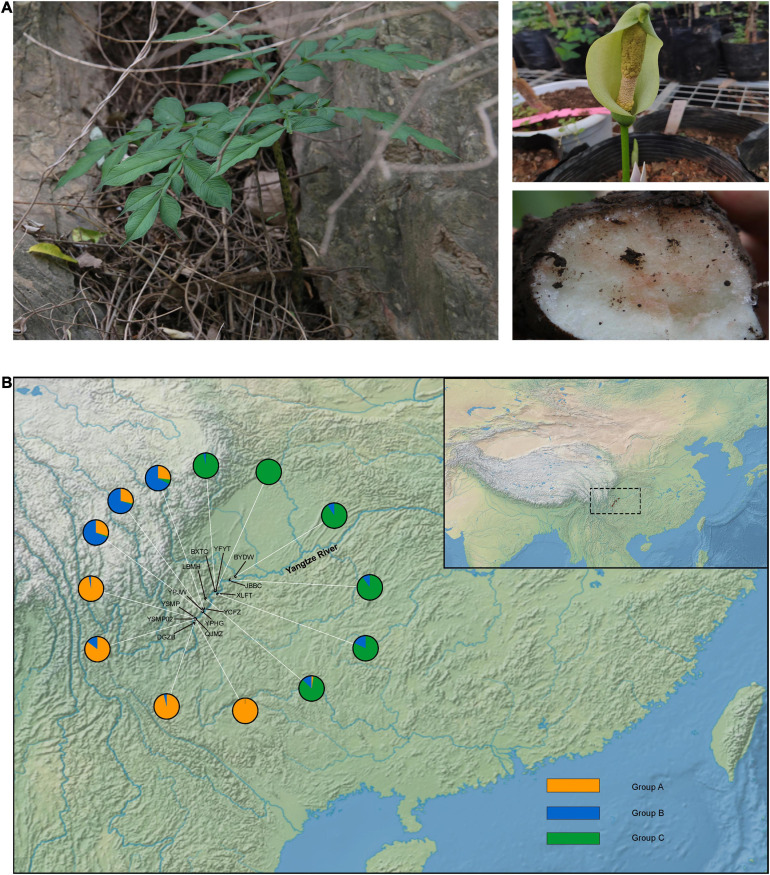
Example and sampling sites of *Amorphophallus albus*. **(A)** The leaf, flower, and corm of *Amorphophallus albus*; **(B)** map of the collection sites of *Amorphophallus albus*, individual pie charts indicate the membership proportions of each population for the inferred number of *K* = 3 by STRUCTURE.

*Amorphophallus* species are characterised by a single leaf and an underground stem (Figure 1A), and are important sources of konjac glucomannan (KGM) (Chua et al., 2010; Yin et al., 2019). Early investigations show that KGM is a multi-purpose material, with uses as a gelling agent, thickener, film former, and emulsifier (Nishinari et al., 1992; Hu et al., 2006). KGM has also been reported to have the ability to control blood cholesterol and sugar levels, help with weight loss and promote intestinal activity (Zhang et al., 2005; Hu et al., 2006). In the tropical and subtropical regions of Asia, the diverse species of *Amorphophallus* have been historically used as a food source as well as in traditional medicine (Gille et al., 2011). *Amorphophallus* species have been cultivated in China for nearly 2,000 years. *A. albus* is endemic to China, with a native distribution along the upstream region of the Yangtze River (the Jinshajiang River) (Li et al., 2010). *A. albus* has been cultivated by the farmers in this region, where it is harvested for its high KGM content (Zhang et al., 1999). The cultivation of this plant is so recent that no comprehensive breeding programs have been conducted; instead, local farmers simply collected tubers from wild populations and have grown them in their home gardens for many generations (Yin et al., 2019).

In cultivation, *A. albus* is an asexually reproduced crop that can be harvested 2–3 years after planting. *A. albus* can propagate several cormels in one growth cycle, and farmers usually harvest swollen corms and leave cormels in the soil as the “seed” for next year (Tang et al., 2020). Compared to other crops (e.g., rice, wheat) with a domestication history of nearly 10,000 years, the domestication of *A. albus* is relatively short (less than 2,000 years) (Liu, 2003; Zhou et al., 2017). Although *A. albus* has a narrow range along the downstream regions of the Jinshajiang River, heterogeneous micro-environments exist in this area due to the diverse geographic features of the Yunnan–Guizhou Plateau. Considering its short domestication time and limited distribution, *A. albus* offers an excellent model for evaluating genetic differentiation and local adaptions for crops in the early stages of domestication.

The agricultural domestication process, which allows for the crop’s widespread cultivation, often requires substantial adaptation, including for traits related growth, water use, and climate (Aslan et al., 2015). A deep understanding of the genetic response to abiotic selective pressure and of the genes involved in adaption to climate is essential for broadening the genetic base of the cultivar (Singh, 2001). After years of natural selection and domestication, *A. albus* has formed stable variations in different cultivation areas, leaving local germplasm with specific leaf colors, leaf surface spots, and content of the KGM (Srzednicki and Borompichaichartkul, 2020). Our previous population genetics study, based on 17 simple sequence repeat (SSR) markers, suggested that *A. albus* germplasms should be divided into two genetic groups corresponding to geographic distribution (Yin et al., 2019). However, the study relied on only a few SSRs, which lacked the power to infer fine-scale genetic patterns and functional genomic adaptation in *A. albus*. Besides geographic factors, what role has the heterogeneous climate played in shaping the differentiation of *A. albus* germplasms? Next generation sequencing (NGS) has revolutionised genetic studies of population differentiation and adaptive selection, making it possible to study these phenomena in a wide range of species (Henry, 2012). In order to further investigate population differentiation and its potential environmental drivers in *A. albus*, we used restriction site-associated DNA sequencing (RAD-seq) to generate genome-wide SNPs and conducted population genetics analyses as well as genome-environment associations (GEAs) of *A. albus*. The goal of our study was to: (1) describe the genome-wide genetic diversity pattern in *A. albus*; (2) evaluate the relative effects of geographic and environmental factors in shaping genetic differentiation of *A. albus* populations; (3) find genomic regions which might be associated with local adaption in *A. albus*. These results will shed light on the domestication history and patterns of selection during the early stages of domestication in *A. albus*, which could accelerate crop improvement in the future breeding programs.

## Materials and Methods

### DNA Sampling and Genotyping

A total of 87 individuals from 13 local cultivated populations of *A. albus* landrace germplasms (2–8 individuals per population) were collected during 2016 and 2017 (Figure 1 and Table 1). Each individual was randomly selected from sampling sites, and the genomic DNA was extracted from the leaves using a commercial DNA isolation kit (Tiangen, Beijing, and China). DNA samples were digested with EcoRI enzyme, randomly fragmented and prepared as multiplexed RAD libraries following the established methods (Baird et al., 2008). Samples were pair-end sequenced with a read length of 150 bp on a NovaSeq platform (Illumina, San Diego, CA, United States).

**TABLE 1:** The statistical values of genetic diversity within populations from variant and all positions data of RAD-seq.

**Population**	***N***	***A*_P_**	**Percent of polymorphic loci (%)**	***H*_O_**		***H*_E_**		**π**		***F*_IS_**	
				**Variant positions**	**All positions**	**Variant positions**	**All positions**	**Variant positions**	**All positions**	**Variant positions**	**All positions**
YPHG	3	1	0.0152	0.1072	0.00007	0.0829	0.00006	0.0994	0.00007	−0.0135	−0.00001
YPJW	5	12	0.0239	0.1456	0.00011	0.1154	0.00008	0.1296	0.00009	−0.0317	−0.00002
YSMP02	2	–	–	–	–	–	–	–	–	–	–
JBBC	8	50	0.0260	0.1515	0.00011	0.1117	0.00008	0.1197	0.00009	−0.0504	−0.00004
XLFT	8	48	0.0265	0.1739	0.00013	0.1202	0.00009	0.1288	0.00009	−0.0820	−0.00006
DGZB	8	36	0.0270	0.1943	0.00014	0.1373	0.0001	0.1471	0.00011	−0.0911	−0.00007
QJMZ	8	81	0.0262	0.1815	0.00013	0.1270	0.00009	0.1361	0.0001	−0.0930	−0.00007
YSMP	8	62	0.0323	0.1483	0.00011	0.1383	0.0001	0.1484	0.00011	−0.0027	0
YCFZ	8	17	0.0288	0.1465	0.00011	0.1258	0.00009	0.1349	0.0001	−0.0247	−0.00002
LBMH	8	245	0.0359	0.1331	0.0001	0.1417	0.0001	0.1517	0.00011	0.0460	0.00003
BXTC	8	35	0.0271	0.1647	0.00012	0.1281	0.00009	0.1374	0.0001	−0.0555	−0.00004
BYDW	8	15	0.0282	0.1526	0.00011	0.1281	0.00009	0.1373	0.0001	−0.0328	−0.00002
YFYT	5	17	0.0136	0.1510	0.00011	0.0825	0.00006	0.0928	0.00007	−0.1046	−0.00008

*N, number of individuals; A_P_, private allele number; H_O_, observed heterozygosity; H_E_, expected heterozygosity; π, nucleotide diversity; F_IS_, inbreeding coefficient.*

Prior to assembly, the raw data of each sample underwent quality control by removing adapters and paired reads with alternative reads containing more than 50% low-quality bases (*Q*-value ≤ 5) or 10% unidentified nucleotides (Ns). Stacks v2.3b was then used to assemble pair-ended reads and identify SNPs (Rochette and Catchen, 2017). We set the minimum depth of coverage to create a stack at three sequences and the maximum number of different nucleotides allowed between catalog loci as two nucleotides. The SNP loci were filtered with the “populations” software implemented in Stacks v2.3b (Rochette and Catchen, 2017). To obtain high-quality SNPs, we removed loci with a global minor allele frequency (MAF) less than 0.05 or that were missing in more than 15% of total samples. We retained only loci that were present in at least 80% of the individuals from the population in at least 11 populations. Only the first SNP per locus was retained to avoid linkage bias.

### Analyses of Population Genetic Diversity and Differentiation

Population genetic diversity statistics, including observed heterozygosity (*H*_*O*_), expected heterozygosity (*H*_*E*_), nucleotide diversity (π), and inbreeding coefficient (*F*_*IS*_) were calculated for variant loci as well as all nucleotide positions using the “populations” program in Stacks v2.3b (Rochette and Catchen, 2017). Population YSMP02, which was comprised of fewer than three samples, was excluded from all genetic diversity analyses. Population differentiation (*F*_*ST*_) among 13 populations of *A. albus* was analysed with the “populations” program and the significance was inferred by Arlequin 3.5 with 1,000 permutations (Excoffier and Lischer, 2010).

### Genetic Structure of *A. albus* Populations

Genetic structure of 13 *A. albus* populations was assessed in multiple ways. First, we used a Bayesian clustering approach implemented in STRUCTURE 2.3.4 (Pritchard et al., 2000). We set the possible cluster numbers (*K*) ranging from 1 to 10, and 10 independent calculations were run for each *K* number. The simulations were conducted with 100,000 burn-ins as well as 500,000 Markov Chain Monte Carlo (MCMC) chains after burn-ins. The most likely cluster number was determined by the Δ*K* value calculated by Structure Harvester 0.6 (Earl and vonHoldt, 2012). In addition to STRUCTURE, principal component analysis (PCA), and fineRADstructure (Milan et al., 2018) were used as complementary assessments of population structure. The PCA plot of *A. albus* germplasms was drawn using the “adegenet” package in R (Jombart, 2008). The fineRADstructure software is a modified version of the fineSTRUCTURE package (Lawson et al., 2012), which uses haplotype linkage information to calculate a co-ancestry matrix based on the most recent coalescence among sampled individuals (Milan et al., 2018). The input file was generated from the output of Stacks “populations” program using the data conversion script provided by fineRADstructure (Milan et al., 2018). The fineRADstructure pipeline was run with 100,000 burn-ins and 100,000 MCMC chains, sampling every 1,000 steps. A tree illustrating the relationships among all individuals was also constructed with 10,000 hill-climbing iterations.

To quantify the genetic variance distributed in different hierarchical levels of *A. albus* germplasms, we assigned the 13 populations into genetic groups according to genetic structure analyses. Analysis of molecular variance (AMOVA) implemented in Arlequin 3.5 was used to detect the genetic variance among genetic clusters, among populations within clusters and within populations. The significance was calculated with 9,999 permutations (Excoffier and Lischer, 2010).

### Genetic, Geographic, and Environmental Correlations

To assess the contributions of geography and environment in driving the genetic differentiation of *A. albus*, mantel tests of isolation by distance (IBD) and isolation by environment (IBE) were conducted. Geographic distance between each pair of populations was calculated with the geographical coordinates of each population using GenAlex 6.5 (Peakall and Smouse, 2012). Using GPS coordinates of sampled populations, 19 climate variables with a resolution of 2.5 min were downloaded from the WorldClim^1^ (Supplementary Table 1). The 19 climatic variables were first subject to PCA using SPSS Statistics 17.0 to reduce dimensionality. PCA suggested that the first three principal components (PCs) explained 99.19% of the total variation (Supplementary Table 2). We therefore selected the first three PCs of climatic variables to use as data points, and calculated Euclidean distance using “ecodist” package in R to create a pairwise environmental distance matrix for all populations (Goslee and Urban, 2007). Finally, the genetic, geographic, and environmental correlations were tested using GenAlex 6.5 with 1,000 permutations (Peakall and Smouse, 2012).

We used redundancy analyses (RDA), implemented in the “vegan” package in R, to simultaneously estimate the effects of geography and environment on genomic variation (Oksanen et al., 2015). The genomic SNPs from each individual were treated as response factors, and geographic (latitude and longitude) and climatic (three climatic PCs) variables were used as explanatory factors. An initial global test was conducted using 999 permutations to determine whether the genome-wide SNP variation of 87 samples could be explained by geography and climatic factors. To determine the role of each variable separately, a partial RDA (with 999 permutations) which the effects of all but the tested explanatory variables were treated as conditioned factors was tested one by one (Wang et al., 2017).

### Genome-Wide Signatures of Adaptive Differentiation

We used BayPass 2.1 to conduct the genome scan for adaptive divergence in *A. albus* germplasms (Gautier, 2015). This software identifies overly differentiated outliers by calculating the *XtX* statistics and the matrix Ω (Günther and Coop, 2013; Gautier, 2015; Flori et al., 2019). First, the *XtX* statistics of *A. albus* germplasms were calculated based on genomic SNPs. Then, a pseudo-observed data set (POD) with 10,000 SNPs was simulated according to parameters estimated from the real data set and used to calibrate the *XtX* statistics (Gautier, 2015). The posterior estimate of Ω obtained with the POD was compared with that obtained from the real data. Finally, a 1% significance threshold of the *XtX* statistics was used to identify genomic footprints of selection.

To complement the outlier tests, we also carried out GEA analyses using BayPass 2.1, with the AUX model and parameters estimated above (Gautier, 2015). We treated three PCs of the climate variables as covariables, and the significant association of each SNP with each covariable was judged by a calibrated Bayes Factor (BF) of POD with 1% threshold.

## Results

### RAD-Seq Assembling and Genotyping

Sequencing RAD-Tags from 87 individuals of *A. albus* germplasms produced 453.75 GB of clean data. The genomic data per sample ranged from 2.97 to 10.65 GB, with the average value being 5.22 GB (Supplementary Table 3). The minimum and maximum coverage depth of each sample was 7.26 and 13.92, respectively, and the average depth was 9.12 (Supplementary Table 3). After applying the assembly and filtering criteria described above, a final SNP data set consisting of 24,225 loci was retained for subsequent analyses.

### Genetic Diversity and Differentiation Among Populations

Genetic diversity indices, including *H*_*E*_ and π, were highest in population LBMH and lowest in YFYT, when calculated from variant loci and from all positions (Table 1). Meanwhile, the observed heterozygosity (*H*_*O*_) within populations was highest in DGZB and lowest in YPHG. The largest number of private alleles was found in LBMH (Table 1). Pairwise *F*_*ST*_ values among populations ranged from 0.038 (YCFZ versus YPJW) to 0.352 (YFYT versus YPHG), and significant genetic differentiations were detected between most population pairs (69 out of 78 *F*_*ST*_ values) (*P* < 0.05) (Table 2).

**TABLE 2:** Pairwise *F*_ST_ values among 13 populations of *Amorphophallus albus* based on 24,225 SNPs.

**Population**	**DGZB**	**QJMZ**	**YSMP**	**YSMP02**	**YPJW**	**YPHG**	**YCFZ**	**LBMH**	**BXTC**	**XLFT**	**JBBC**	**BYDW**
QJMZ	**0.149**											
YSMP	0.101	**0.135**										
YSMP02	0.126	0.108	0.098									
YPJW	**0.219**	**0.239**	**0.202**	0.209								
YPHG	**0.212**	**0.245**	**0.206**	0.257	0.091							
YCFZ	**0.198**	**0.215**	**0.183**	**0.171**	0.038	0.070						
LBMH	**0.217**	**0.233**	**0.186**	**0.188**	**0.175**	**0.175**	**0.153**					
BXTC	**0.251**	**0.269**	**0.217**	**0.225**	**0.209**	**0.215**	**0.179**	**0.109**				
XLFT	**0.257**	**0.274**	**0.222**	**0.231**	**0.218**	**0.225**	**0.185**	**0.128**	**0.134**			
JBBC	**0.275**	**0.292**	**0.238**	**0.251**	**0.233**	**0.243**	**0.200**	**0.126**	**0.122**	**0.147**		
BYDW	**0.247**	**0.266**	**0.212**	**0.223**	**0.205**	**0.212**	**0.175**	**0.096**	**0.108**	**0.112**	**0.104**	
YFYT	**0.330**	**0.349**	**0.281**	**0.346**	**0.303**	**0.352**	**0.245**	**0.163**	**0.137**	**0.195**	**0.175**	**0.168**

*Bold values indicate significant levels (*P* < 0.05).*

### Analysis of Population Genetic Structure

**FIGURE 2:**
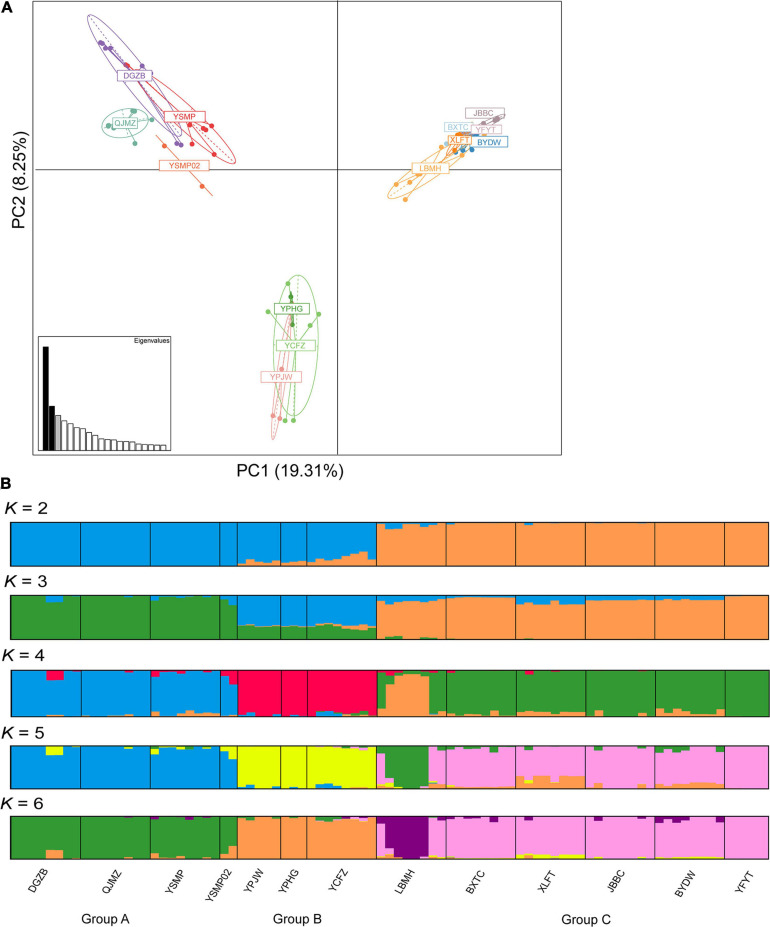
Genetic structure of *Amorphophallus albus* germplasms investigated by principal component analysis **(A)** and STRUCTURE **(B)**.

**FIGURE 3:**
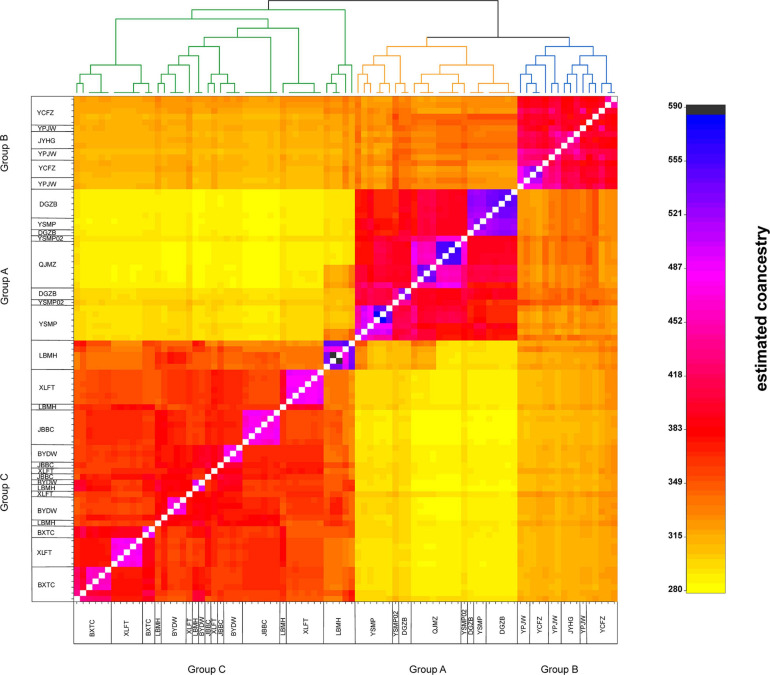
The clustered co-ancestry matrix of *Amorphophallus albus* germplasms by fineRADstructure, indicating pairwise co-ancestry between individuals.

The STRUCTURE analysis indicated that *K* = 6 was the best supported number of genetic clusters, with the second largest Δ*K* value detected when *K* = 2 (Supplementary Figure 1). However, results from the other two analyses, PCA and fineRADstructure, suggested that the 13 populations should been divided into 3 genetic groups. As the result of *K* = 6 was not biologically informative, we selected *K* = 3 as the optimal genetic cluster number (Figure 2B). The three genetic groups were comprised of a geographically upstream group (DGZB, YSMP, YSMP02, and QJMZ; Group A), a midstream group (YPJW, YPHG, and YCFZ; Group B), and a group distributed along the downstream region (LBMH, BXTC, XLFT, JBBC, BYDW, and YFYT; Group C) (Figures 1B, 2B). The 13 populations were essentially grouped by geographic proximity, and substantial genetic introgression was detected in the 3 populations of group B (Figures 1B, 2B). Analysis of fineRADstructure further revealed the hierarchical genetic structure of *A. albus* germplasms. The 13 populations were first divided into 2 groups, with the up-stream group further differentiated into two sub-clusters (Figure 3). The divergence within the upstream group was also supported by an independent STRUCTURE analysis using only the up-stream populations (Supplementary Figure 2).

To quantify the genetic variance distribution in *A. albus* germplasms, scenarios of two and three genetic groups were tested using AMOVA. When assigning populations into two groups, 24.68% of variation was among genetic groups (*P* < 0.0001). When the populations were assigned into three groups, a higher percentage of variation (29.90%) among groups was revealed (*P* < 0.0001) (Table 3).

**TABLE 3:** Results of the analyses of molecular variance (AMOVA) based on SNP datasets.

**Source of variation**	**Variance components**	**Percentage of variation**	***F*-statistics**	***P*-value**
**Two genetic groups**				
Among groups	163.718	24.681	*F*_CT_ = 0.247	*P* < 0.0001
Among populations	129.089	19.461	*F*_SC_ = 0.258	*P* < 0.0001
Within populations	370.520	55.858	*F*_ST_ = 0.441	*P* < 0.0001
**Three genetic groups**				
Among groups	195.672	29.902	*F*_CT_ = 0.299	*P* < 0.0001
Among populations	88.179	13.475	*F*_SC_ = 0.192	*P* < 0.0001
Within populations	370.520	56.622	*F*_ST_ = 0.434	*P* < 0.0001

### Genetic, Geographic, and Environmental Correlations

**FIGURE 4:**
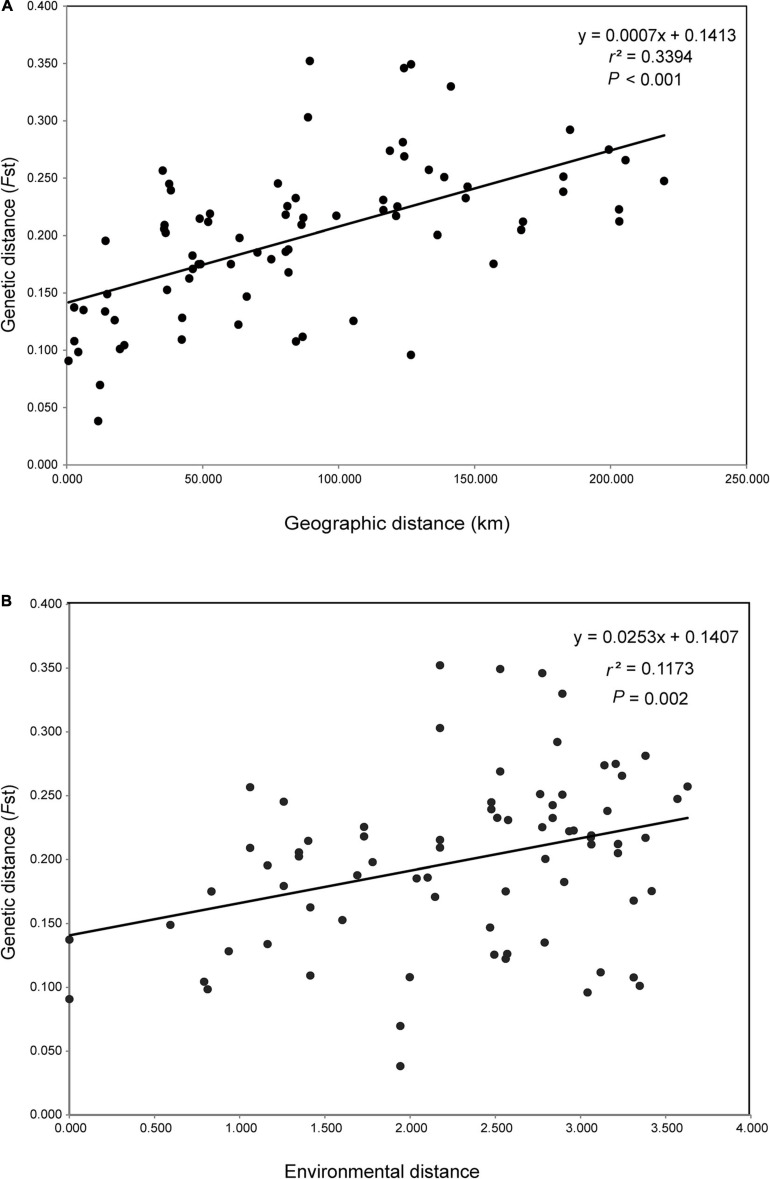
Mantel tests of geographic, environmental, and genetic correlations. **(A)** Correlation of pairwise geographic distance versus pairwise genetic distance (*F*_*ST*_); **(B)** correlation of pairwise environmental distance versus pairwise *F*_*ST*_.

**FIGURE 5:**
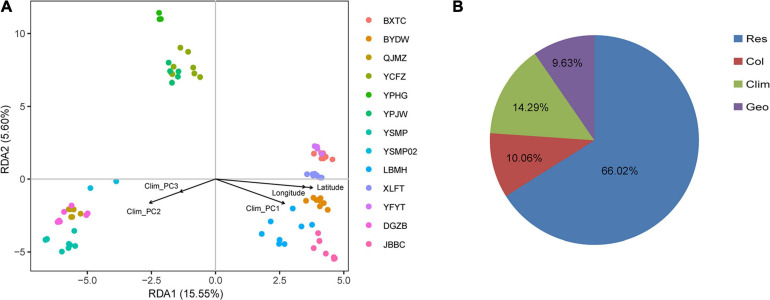
Redundancy analysis (RDA) performed with 24,225 SNPs among 87 samples. **(A)** The first two axes shown for RDA, using latitude, longitude, and climate as constraining variables. Colored points represent samples from different populations. **(B)** Proportion of total SNP variation explained in RDA by climatic variable (Clim), spatial structure (Geo) or their collinear effect (Col), respectively. The residential variation (Res) was 66.02%.

The first three PCs summarised 58.52, 32.22, and 8.45%, respectively, of the total variation in 19 climatic variables downloaded from WorldClim (Supplementary Table 2). Both IBD and IBE analyses all suggested that the genetic distance was significantly correlated with geographic and environmental distances (Figures 4A,B). The proportion of genetic variation that could be explained by geographic and climatic factors was further assessed with RDA. The initial global RDA test was highly significant, and the five constraining variables recovered 33.98% of the genotypic variance (Supplementary Table 4). The first two axes of RDA explained 15.55 and 5.60% of the total variance (Figure 5A). When testing the role of each variable in predicting genotypic variation independently, all partial RDA tests revealed a significant effect for each geographic and climatic factor by conditioning all other variables (Supplementary Table 4). Overall, the climatic variables explained a higher percent of SNP variation (14.29%) than geographic factors (9.63%) (Figure 5B).

### Genome-Wide Footprints of Adaptive Differentiation

For the simulations in BayPass, the Spearman’s correlation between the posterior estimate of Ω obtained with the POD and that acquired from the real data set was nearly one (ρ = 0.9988) (Supplementary Figure 3). This indicated that the POD faithfully mimics the real data set, and the 1% significance threshold of the *XtX* statistics (*XtX* = 19.62) was robust to identify outliers under genomic selection. Through this genome scan for outlier loci, 20 outliers were detected by the *XtX* calibration in BayPass (Supplementary Figure 4A and Supplementary Table 5). GEA analyses revealed that 14 loci were significantly associated with Clim_PC1, 14 loci with Clim_PC2, and 22 loci with Clim_PC3 (Supplementary Figures 4B–D and Supplementary Table 5). In total, 20 loci were detected as significant in two or more tests (Supplementary Table 5).

## Discussion

### Genetic Diversity and Geographical Differentiation Within *A. albus*

In this study, we analysed the population genetic diversity of *A. albus* germplasms with genome-wide SNPs. Overall, in our study the highest genetic diversity indices, such as the number of private alleles, expected heterozygosity and nucleotide diversity, were all detected in the LBMH population. Future breeding strategies may seek to give a high priority to this diverse population. We found that the genetic diversity revealed by SNPs (*H*_*E*_: 0.083–0.142) is lower than that evaluated from SSRs (*H*_*E*_: 0.294–0.487) (Yin et al., 2019). While SNP markers are bi-allelic, SSR markers are multi-allelic (Cantelmo et al., 2016), which likely increases rates of heterozygosity and allelic richness at these loci. For example, Fischer et al. (2017) compared the effects of microsatellite and SNPs in estimating genomic diversity of *Arabidopsis halleri*, and also found that *H*_*E*_ indices estimated from SNPs were overall lower than those estimated from SSRs. They suggested that a few SSR markers therefore could not adequately reflect genome-wide genetic diversity due to the small number of markers used, ascertainment bias for hypervariable loci and the high variance in SSR-derived diversity estimates (Fischer et al., 2017). In light of this, the previous microsatellite-based heterozygosity estimates of *A. albus* germplasms might be biased. On the other hand, NGS methods like RAD-seq provide an opportunity to explore genetic diversity in plants on a much larger scale than was possible with earlier technologies, and allows even the most complex plant genomes to be tackled (Ganal et al., 2009; Henry, 2012; Bolger et al., 2014).

Our results supported the previous inference from SSR makers that *A. albus* genetically differentiated into two geographic groups, one from the upstream region and another (Group C) in the downstream region (Figure 1B). A novel result from the current study was the identification of sub-divergence within the upstream group, which can be separated into two genetic clusters (Figure 2). Although the plot of Δ*K* value indicate that *K* = 6 was the most supported genetic cluster number in the STRUCTURE analysis, the result of *K* = 6 was not biologically informative. The other two analyses (PCA and fineRADstructure) all suggested that populations of *A. albus* germplasms should be divided into three genetic groups (Figure 2B). We also conducted an independent STRUCTURE analysis of only the seven populations from the upstream region, which supported the genetic division of the upstream group (Supplementary Figure 2). All these tests indicate that the inference about genetic relationships among populations was robust. Our previous study only detected the two genetic clusters. Because of both an increase in the number of independent loci and in the overall number of alleles, the thousands of SNPs outperformed the few SSRs in the genetic assignment analysis (Fischer et al., 2017). Our study clearly demonstrates the power of genome-wide SNPs in discovering cryptic genetic structure of domesticated plant.

### Drivers Shaping the Geographic Differentiation Pattern of *A. albus*

Much previous work has been done to investigate the role of geographic and environmental factors in driving genetic patterns of natural populations (e.g., Orsini et al., 2013; Wang et al., 2017). Our results, based on separate IBD and IBE analyses, indicated that the genetic differentiation of *A. albus* was shaped by both effects of IBD and IBE. The existence of IBD in natural populations is usually interpreted as the equilibrium between random drift and migration, in which gene flow is dependent on geographical distance (Gao et al., 2015). This pattern can be generated by both island and stepping-stone models (Orsini et al., 2013). Because stepping-stone colonisation should result in lower genetic diversity in populations that were colonised later, we could potentially distinguish these two processes (Prunier and Holsinger, 2010). Given the short domestication time of *A. albus*, the genetic pattern of germplasms should not yet be extensively affected by cultivation of this species. As no clear variation pattern of genetic diversity was observed in 13 populations, we conclude that the geographically isolated landscapes contribute to the genetic differentiation pattern of *A. albus*.

We also observed a significant effect of climatic factor distances on genetic differentiation of *A. albus*. A large body of genetic research has identified IBE as one driver of population differentiation, highlighting the importance of environmental adaptation in shaping genetic differentiation patterns. RDA analysis enables identification and testing of the effect of individual variables influencing genomic variability, while also offering the ability to detect collinearity between them (Lasky et al., 2012; Wang et al., 2017). Our RDA results supported a stronger influence of environmental adaptation over geographic factors in driving population differentiation, with 14.29% of genetic differentiation being explained by the environment (Figure 5B). That the environment was more important than geography in predicting genetic differentiation suggests a predominant role of environmental adaptation in *A. albus*. Given the heterogeneous micro-environments in this region, these populations might have adapted to the local conditions of their microhabitats. As reported in other species (Ye et al., 2016), geographic isolation may have induced initial differentiation, which was then reinforced by adaptation in response to local climate and environmental factors.

### Genomic Regions Associated With Local Adaptation

Understanding genetic variation for local adaption in the gene pool of domesticated landraces is important for the selection of crops with increased yield potential in future environments (Redden, 2013). In this study, BayPass software identified highly differentiated SNPs based on a calibration procedure of the XtX statistic (Günther and Coop, 2013) by correcting for confounding demographic effects (Gautier, 2015). In total, 20 loci were detected as under positive selection pressure with this method. Additionally, more than 20 loci in total were significantly associated with climate factors as revealed by GEA (Supplementary Figures 4B–D and Supplementary Table 5). These environment-associated SNPs might reflect the impact of local adaptation, which has been implicated in studies of other species (Lotterhos and Whitlock, 2014; Wang et al., 2017). Previous studies of local adaptation have shown a trend toward complex adaptation involving many loci, each under relatively weak selection (Allaby et al., 2015; Zhou et al., 2017). The multiple loci identified in our study support the idea of complex local adaptation in *A. albus* populations. Due to the lack of a reference genome, we could not identify the genomic distribution of the identified loci nor linked functional genes. Despite this knowledge gap, the conservation of these adaptive genomic regions can be included in breeding plans for this species that would lead to robust and locally adapted cultivars (Zhou et al., 2017).

### Breeding Implications

Although *A. albus* has been cultivated and consumed for a long time in China, the crop is cultivated from unimproved original genetic material in most regions (Srzednicki and Borompichaichartkul, 2020). The reproduction ratio of *Amorphophallus* species is much lower compared with most other tuber crops. For example, the annual reproduction coefficient of potatoes, sweet potatoes, and other tuber crops can be up to more than a dozen times, but that of *A. albus* is only one third of these plants (Adams and Stevenson, 1990; Srzednicki and Borompichaichartkul, 2020). The lack of seed corms further hindered the selection on agronomic traits (Yin et al., 2019).

Sexual crossbreeding should be carried out on *A. albus* to offset its demerit of a low yield (Srzednicki and Borompichaichartkul, 2020). The crossbreeding attempts of this plant have just started in last 20 years. The genomic variation pattern of *A. albus* observed in this study can guide breeding programs. Based on the relatively low genetic diversity, more breeding material is needed. When making crossbreeding strategies, the implement of materials from different genetic groups should be considered. Only in this way can the later generations have greater variation and, therefore, provide more selection opportunities.

In conclusion, we described the genomic genetic diversity of *A. albus* germplasms using tens of thousands SNPs developed from RAD-seq. Cryptic genetic structure was also uncovered in this species, which demonstrated the increased power of genomic SNPs over traditional markers. We further analysed the roles of geographic and environmental factors in shaping genetic patterns of *A. albus* germplasms, and discovered genomic regions that might be associated with local adaptation. Together these findings shed light on the geographic differentiation patterns of *A. albus*, and will inform the future breeding programs of this agriculturally important plant.

## Data Availability Statement

The datasets presented in this study can be found in online repositories. The names of the repository/repositories and accession number(s) can be found below: https://www.ncbi.nlm.nih.gov/, PRJNA715021.

## Author Contributions

YG, LT, and DD conceived and designed the study. YG, HChu, HChen, and SY collected the samples. SY and YZ carried out the laboratory work and performed the analyses. YG drafted the manuscript. All authors contributed to writing and editing and read and approved the final manuscript.

## Conflict of Interest

The authors declare that the research was conducted in the absence of any commercial or financial relationships that could be construed as a potential conflict of interest.

## Publisher’s Note

All claims expressed in this article are solely those of the authors and do not necessarily represent those of their affiliated organizations, or those of the publisher, the editors and the reviewers. Any product that may be evaluated in this article, or claim that may be made by its manufacturer, is not guaranteed or endorsed by the publisher.

## Footnotes

1 https://www.worldclim.org/
