# Supplementary material for: Genome-Wide SNPs Provide Insights on the Cryptic Genetic Structure and Signatures of Climate Adaption in Amorphophallus albus Germplasms
Source: Front Plant Sci. 2021 Jul 23;12:683422. doi: 10.3389/fpls.2021.683422 (PMC8343094; doi:10.3389/fpls.2021.683422)
Supplement: Supplementary file 1 [file Data_Sheet_1.DOCX]

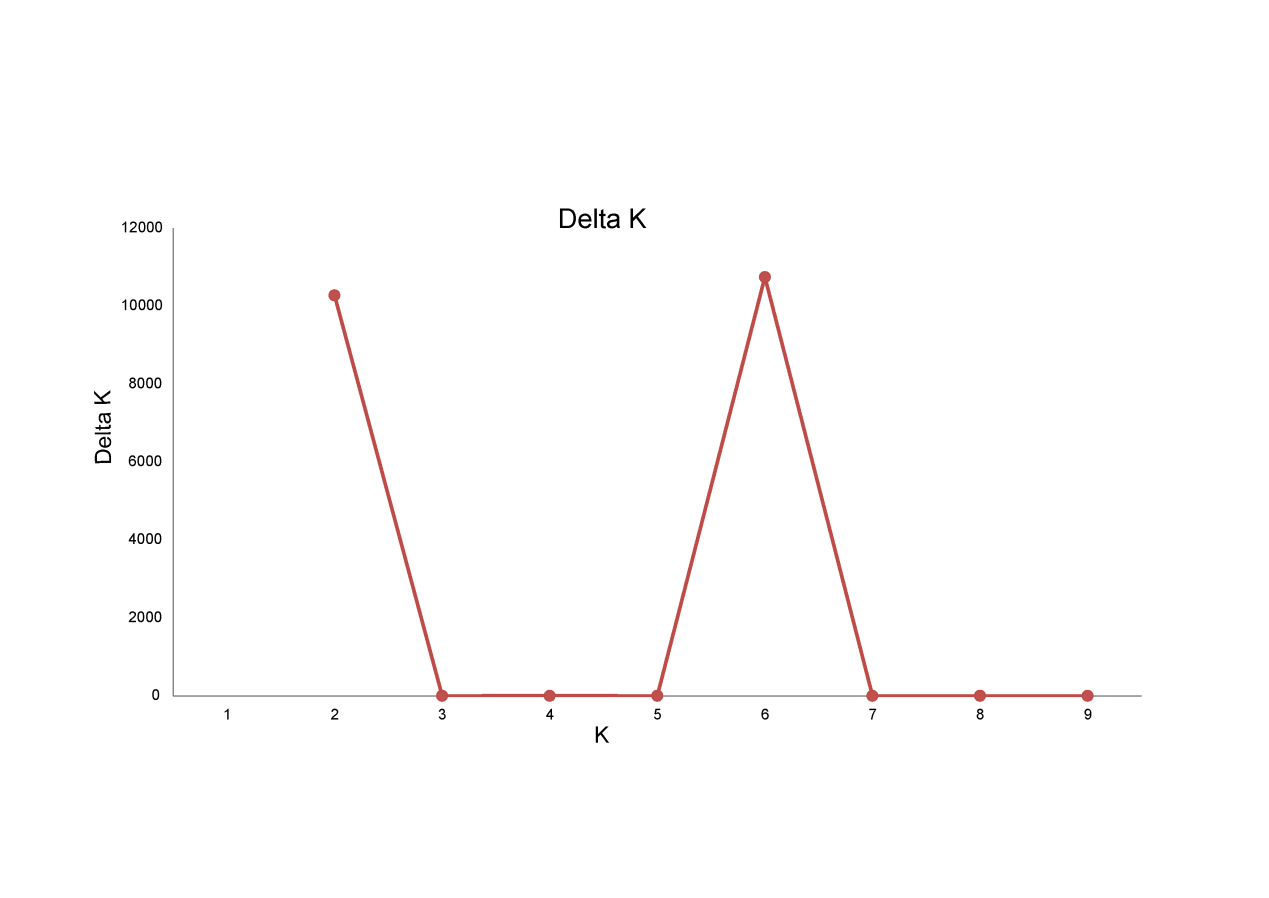


**Figure S1** Δ*K* estimates of the posterior probability distribution for the STRUCTURE analysis based on 13 populations of *Amorphophallus albus*.


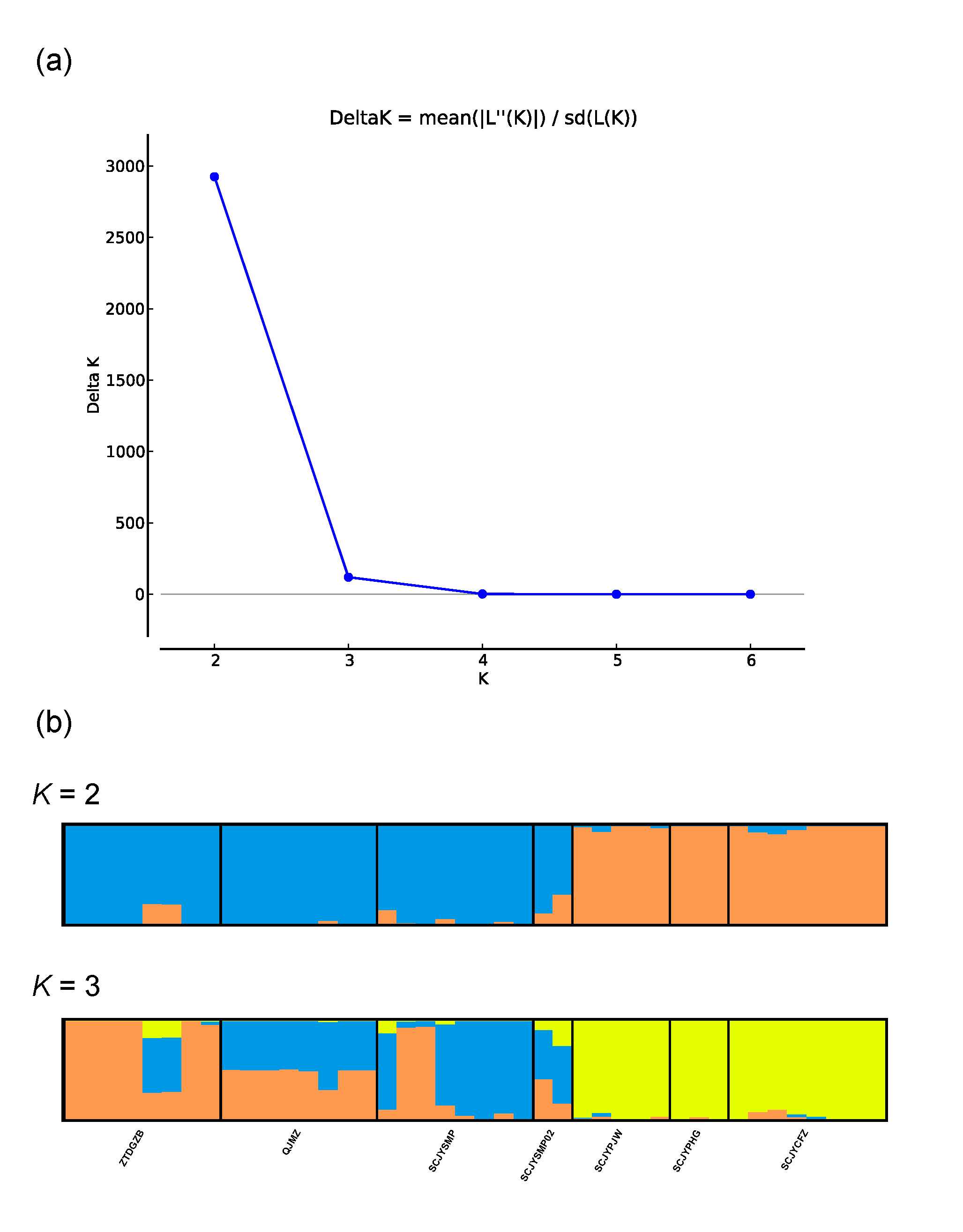


**Figure S2** The STRUCTURE analysis of seven *Amorphophallus albus* populations distributed along the up-stream region of Jinsha River. (a) Δ*K* estimates of the posterior probability distribution. (b) Estimated population structure of *Amorphophallus konjac* populations with *K* =2 and *K* =3.


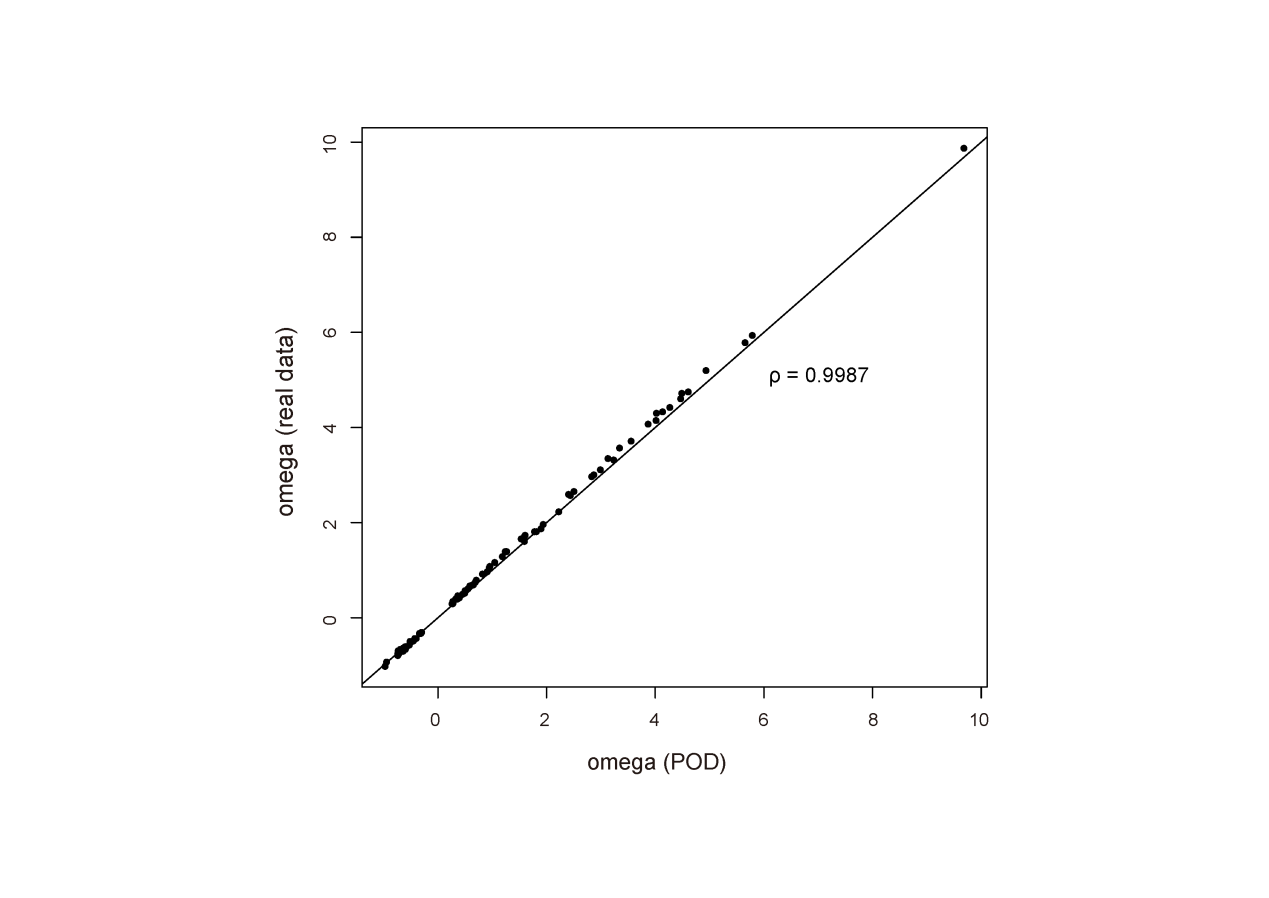


**Figure S3** Plot of the Spearman’s correlation between the posterior estimate of Ω obtained with the pseudo-observed data set (POD) and that acquired from the real data set by BayPass.


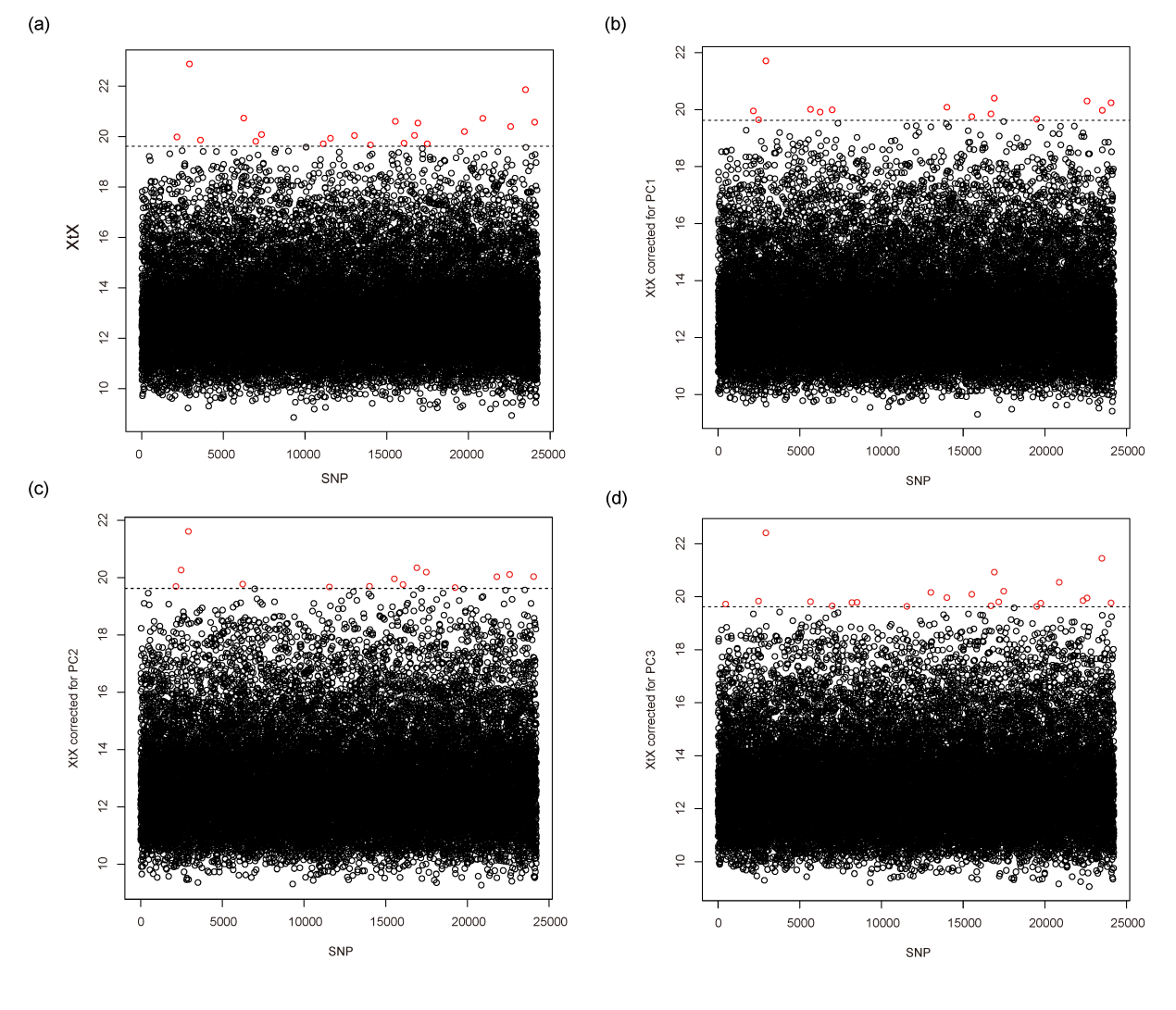


**Figure S4** The XtX-based outliers detection (a) and environment-SNP association analyses (b, c and d) by the program BayPass. Red dot indicates the significant locus.
